# Cytokine Profiling among Children with Multisystem Inflammatory Syndrome versus Simple COVID-19 Infection: A Study from Northwest Saudi Arabia

**DOI:** 10.3390/biology11070946

**Published:** 2022-06-21

**Authors:** Hany M. Abo-Haded, Amer M. Alshengeti, Abdulsalam D. Alawfi, Saad Q. Khoshhal, Khalid M. Al-Harbi, Mohammad D. Allugmani, Dina S. El-Agamy

**Affiliations:** 1 Department of Pediatrics, College of Medicine, Taibah University, Madinah 30001, Saudi Arabia; amshengeti@taibahu.edu.sa (A.M.A.); adawfi@taibahu.edu.sa (A.D.A.); skhoshhal@taibahu.edu.sa (S.Q.K.); kalharbi@taibahu.edu.sa (K.M.A.-H.); 2 Department of Pediatrics, Faculty of Medicine, Mansoura University, Mansoura 35516, Egypt; 3 Department of Infection Prevention and Control, Prince Mohammed Bin Abdulaziz Hospital, National Guard Health Affairs, Madinah 30001, Saudi Arabia; 4 Pediatric Cardiology Unit, Department of Pediatrics, Maternity and Children Hospital (MCH), Madinah 30001, Saudi Arabia; malluqmani@moh.gov.sa; 5 Department of Pharmacology, College of Pharmacy, Taibah University, Madinah 30001, Saudi Arabia; dagamiabdalla@taibahu.edu.sa; 6 Department of Pharmacology, Faculty of Pharmacy, Mansoura University, Mansoura 35516, Egypt

**Keywords:** multisystem inflammatory syndrome, children, cytokine storm, progression, outcome

## Abstract

**Simple Summary:**

Multisystem Inflammatory Syndrome in Children (MIS-C) is a novel syndrome associated with COVID-19. Its manifestations vary from asymptomatic to life-threatening disease. Cytokines are essential mediators of the inflammatory response during MIS-C. In this study, we analyzed the expression of inflammatory markers and cytokines in blood, reported the important clinical characteristics, and correlated these results with short- and mid-term outcomes. Significantly elevated levels of cytokines (IL-1β, IL-6, and GM-CSF) confirmed their role in the severity of manifestations, disease progression, and outcome. Thus, this is one of the earliest studies in Saudi Arabia to evaluate the inflammatory response (cytokine profile) of MIS-C, correlating the clinical phenomena.

**Abstract:**

Background: Multisystem Inflammatory Syndrome in Children (MIS-C) is a novel syndrome associated with severe acute respiratory syndrome coronavirus 2 (SARS-CoV-2) infection with varying clinical features. This study aimed to analyze the expression profiles of cytokines in blood, report the important clinical characteristics, and correlate these with the short- and mid-term outcomes. Methods: This cross-sectional study was conducted on hospitalized children with MIS-C from March 2021 to May 2022. Phenotypes were classified into two groups (A,B) according to the severity of the disease and the need for invasive respiratory support. Clinical features, laboratory parameters, and outcomes were reported. Results: We identified 60 children with MIS-C (mean age of 7.4 ± 3.8 years) compared to 30 age- and sex-matched controls with simple COVID-19. The clinical manifestations of MIS-C patients were fever (100%), respiratory (83.3%), GIT (80%), and conjunctivitis (80%). Twenty-seven MIS-C children (45%) required PICU admission due to shock and needed mechanical ventilation. Anemia, lymphopenia, and elevated levels of inflammatory and tissue injury markers were observed in the MIS-C groups (mainly B). High cytokine levels (IL-1β, IL-6, IFN-α, GM-CSF, and HMGB1) were observed acutely in the MIS-C children, and a persistent elevation of some cytokines were reported at midterm follow-up, especially in Group B. Conclusion: Robust inflammatory response to COVID-19 disease with elevated IL-1β, IL-6, and GM-CSF levels might explain the severity and outcome of the clinical syndrome.

## 1. Introduction

In March 2020, the World Health Organization (WHO) declared the coronavirus disease 2019 (COVID-19) to be a pandemic [1]. COVID-19 is caused by severe acute respiratory syndrome coronavirus 2 (SARS-CoV-2), which is currently infecting millions of people; thousands of people around the world are dying from it [2]. The manifestations of COVID-19 infection range from asymptomatic or mild infection—in most patients—to severe complications that can eventually lead to death. SARS-CoV-2 can trigger both innate and adaptive immune systems, which cause the release of cytokines and chemokines. This response might lead to massive systemic inflammation, which is described as “cytokine storm” [3]. This intense cytokine secretion plays a major role in disease progression, multiple organ damage, and ultimately death [4].

Children represent 15–20% of all COVID-19 cases, and the mortality among them is low compared to that in adults [5]. However, in late April 2020, with the peak COVID-19 caseload, alerts were issued across Europe due to the unexpected emergence of a novel inflammatory syndrome in children that was named Multisystem Inflammatory Syndrome in Children (MIS-C) by the Centers for Disease Control (CDC) [6,7,8]. These children critically present with persistent fever, shock, coagulopathy, and clinical features similar to bacterial sepsis and multi-organ failure [9,10,11,12].

The reported higher inflammatory markers in these children with MIS-C explains the important role of the cytokine storm in pathogenesis and disease progression [13,14,15]. 

Understanding the factors associated with more severe outcomes in MIS-C patients may help in predicting the prognosis and facilitate early treatment decisions [16]. We aimed to investigate and compare the expression profiles of inflammatory markers and cytokines among children with different severities of MIS-C during the first two weeks of their illnesses. Additionally, important clinical manifestations and outcomes of these patients are described.

## 2. Materials and Methods

### 2.1. Study Design and Participants

This cross-sectional study was conducted between March 2021 and May 2022 at the department of pediatrics of the Maternity and Children Hospital (MCH), Prince Salman Medical City (PSMC), Madinah, Saudi Arabia. The study protocol was approved by the Institutional Review Board at PSMC (21-007). Informed consent from the parents/guardians of the participating children was obtained.

Inclusion criteria included: patients under 18 years of age, with confirmed COVID-19 infection as per polymerase chain reaction (PCR) testing of nasopharyngeal swab specimens; and patients who fulfilled the MIS-C case definition of the WHO and the Royal College of Pediatrics and Child Health (RCPCH) criteria (persistent fever > 38.5 °C, neutrophilia, elevated C-reactive protein (CRP) levels, lymphopenia, and evidence of single- or multi-organ dysfunction (shock, cardiac, respiratory, renal, gastrointestinal, or neurological disorder)) [7,8]. Children known to have systemic inflammatory conditions or infectious diseases (bacterial sepsis, staphylococcal or streptococcal shock syndromes, or infections associated with myocarditis such as enterovirus), or those on medications with known influence on immunological factors were excluded.

The enrolled patients were divided into two groups, based on the severity of the disease: Group A—children with mild MIS-C manifestations who were admitted to the ward and did not need any respiratory support or required only non-invasive respiratory support (e.g., O_2_ mask, nasal cannula); Group B—children with moderate to severe MIS-C manifestations who were admitted to pediatric intensive care unit (PICU) and needed invasive respiratory support (e.g., continuous positive airway pressure (CPAP), mechanical ventilation (MV)). All patients in the study were managed in a standardized way, following a local multidisciplinary guideline according to national and international guidelines for MIS-C management [16,17].

A control group of age- and sex-matched children (*n* = 30) presenting with simple COVID-19 infection were selected; these children were thoroughly evaluated (clinically and laboratory) to ensure that they do not have any MIS-C manifestations, and were discharged on the same day of admission (Day 1). Patients of the control group were oriented about the clinical manifestations related to MIS-C and were provided with emergency hospital phone numbers to report any abnormalities within 14 days of their discharge.

### 2.2. Data Collection

A coded database was created for registering patients’ information (demographic data, clinical charts, medical history, laboratory findings, and outcomes), maintaining confidentiality. Two researchers independently reviewed the data to double-check it.

### 2.3. Biochemical Analysis

Blood samples were collected on Day 1 from all children of the study (including the control group), and on Day 14 of admission from the MIS-C groups (A and B) using standard venipuncture collection techniques. Approximately 3–5 mL of peripheral blood was obtained in EDTA-containing plastic tubes (Becton Dickinson, Franklin Lakes, NJ, USA). Standard international normal ranges were used to decide the cutoff point whether the laboratory markers were raised or not.

#### 2.3.1. Basic Investigations

The complete blood count (CBC), CRP levels, and erythrocyte sedimentation rate (ESR) were estimated.

#### 2.3.2. Serum Indices of Tissue Injury

Serum samples were separated using 2000 rpm/20 min centrifugation. Markers of tissue injury (creatine kinase isoenzyme-MB (CK-MB), troponin-T, lactate dehydrogenase (LDH), ferritin, alanine aminotransferase (ALT), aspartate aminotransferase (AST), serum creatinine, and blood urea nitrogen (BUN)) were estimated using commercially available kits based on the instructions provided by the manufacturer (ELITech, Paris, France; Human, Wiesbaden, Germany; Kamiya Biomedical Co., Tukwila, WA, USA).

#### 2.3.3. Cytokine

Different cytokines in the serum were estimated using human cytokine enzyme linked immunosorbent assay kits according to the manufacturer’s instructions. The estimated cytokines included interferon alpha (IFN-α), interferon gamma (IFN-γ), interleukin 1 beta (IL-1β), interleukin 6 (IL-6), interleukin 8 (IL-8), interleukin 10 (IL-10) (BD Bioscience, San Diego, CA, USA), tumor necrosis factor alpha (TNF-α), granulocyte colony-stimulating factor (G-CSF), granulocyte-macrophage colony-stimulating factor (GM-CSF) (R&D, Minneapolis, MN, USA), high-mobility group box 1 (HMGB1) (Biomatik, Wilmington, DE, USA), and human C-X-C motif chemokine ligand 10 (CXCL10) (Invitrogen, Waltham, MA, USA).

### 2.4. Mid-Term Follow-Up

A scheduled follow-up strategy at Day 14 after the onset of COVID-19 infection was assigned for the MIS-C patients (Groups A and B), including a clinical assessment, disease progression, and outcomes. Electrocardiography and echocardiography were performed for children clinically suspected with cardiac dysfunction. Outpatient clinic appointments were provided to MIS-C patients who were discharged before Day 14 of the onset of COVID-19 infection to document the follow-up clinical and laboratory data.

### 2.5. Statistical Analysis

Continuous variables were expressed as the mean (standard deviation, SD) or median (interquartile range, IQR), according to a normal distribution. Categorical data were described in numbers (percentages). Variables between groups were compared using one-way ANOVA followed by Tukey’s post-test while comparison between the two groups (A and B) at Day 14 was done using a Mann–Whitney *U* test. Statistical significance was set at *p* < 0.05. Analyses were performed using SPSS software 22.0 release, SPSS Inc., Chicago, IL, USA.

## 3. Results

Sixty children with MIS-C manifestations were eligible for inclusion after admission to the hospital compared to 30 children diagnosed to have simple COVID-19 (age- and sex-matched controls). The mean age of the MIS-C population was 7.4 ± 3.8 years (range: 2.7–15.4 years); there were 31 males (51.7%) and 29 females (48.3%). All of them were previously healthy with no comorbidities. There was no obvious statistical significance regarding sex or age difference for the risk of severity of the disease. 

Of the 60 MIS-C patients, 33 children (55%) were admitted to the ward and assigned to Group A; the remaining 27 children (45%) required admission to pediatric intensive care (PICU) during their hospital stay and needed invasive respiratory support and, therefore, were assigned to Group B.

A summary of the demographics, clinical presentations, and basic laboratory workup of the study population and the control group at admission (Day 1) is shown in Table 1.

### 3.1. Clinical Characteristics

All MIS-C patients (100%) presented with persistent fever (>38 °C) few days prior to be diagnosed with MIS-C manifestations (mean 6.1 ± 2.3 days). The other common signs and symptoms included respiratory manifestations (cough or tachypnea or wheezes or distress) in 50 patients (83.3%), GIT manifestations (abdominal pain or diarrhea or vomiting) in 48 patients (80%), erythematous skin rash in nine patients (15%), and shock was present in 22 (36.6%) patients. Other manifestations included conjunctival changes in 48 patients (80%) and lymphadenopathy in five patients (8.3%). The control group showed extremely significant difference when compared to the MIS-C groups (mainly Group B) regarding the respiratory manifestations, shock, heart rate, and respiratory rate (*p* < 0.01). Most patients of Group B were admitted with shock (81.5%) and required fluid resuscitation with crystalloids and vasoactive support. Cardiac dysfunction was reported in three children of this group (11.1%).

As regard the hematologic findings in the MIS-C children, there were predominantly anemia, leukocytosis, lymphopenia, and thrombocytopenia, especially in Group B compared to Group A and controls, with an extremely high statistical difference (*p* < 0.01).

The management policy was a multidisciplinary coordinated approach designed according to the extent of the clinical severity. Children with mild manifestations were treated with supportive measures (antipyretics, fluids), while patients of the severe group were administered IVIG and corticosteroids. Owing to persistence fever in the latter group, about 20% of patients required a repeated dose of IVIG and increased (maximum) dose of steroids. The most required respiratory support was invasive MV for cases presenting hemodynamic instability and shock (22 children, 81.5%).

### 3.2. Laboratory Investigations

**Figure 1:**
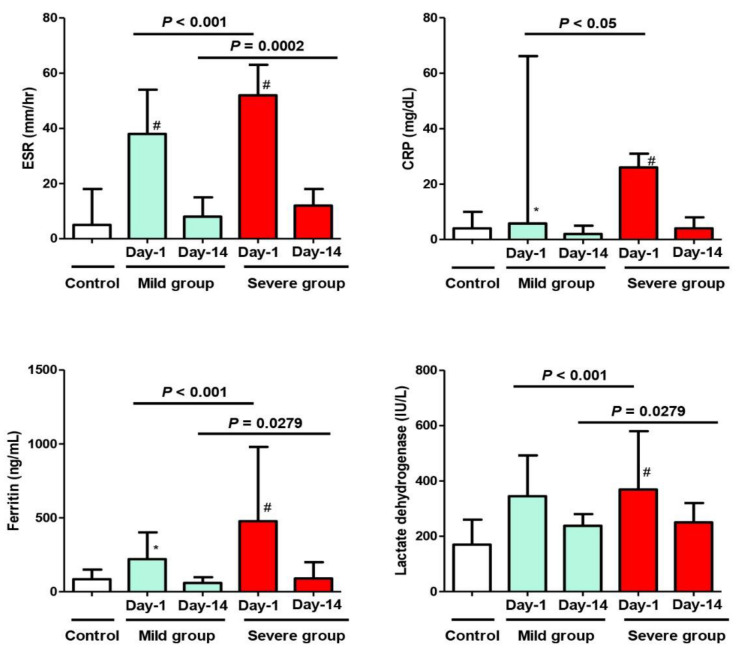
Changes in the inflammatory parameters (ESR, CRP, Ferritin, LDH) of the study cohort on Day 1 and Day 14 after admission with MIS-C manifestations. Control Group = Simple COVID-19 infection; Group A = Mild MIS-C; Group B = Severe MIS-C. ESR: Erythrocyte sedimentation rate; CRP: C-reactive protein; LDH: Lactate dehydrogenase. * *p* < 0.05 and ^#^ *p* < 0.01, statistically significant difference compared to the control group (one-way ANOVA followed by Tukey’s post-test).

**Figure 2:**
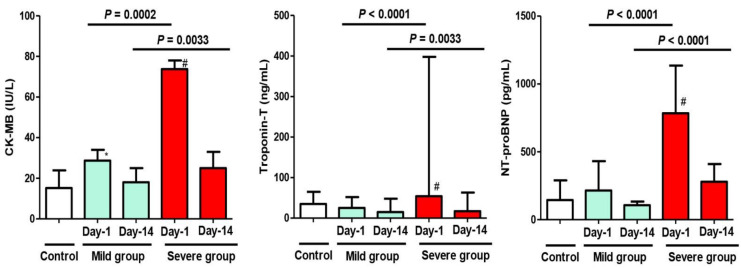
Changes in the cardiac parameters (CK-MB, Troponin T, NT-proBNP) of the study cohort on Day 1 and Day 14 after admission with MIS-C manifestations. Control Group = Simple COVID-19 infection; Group A = Mild MIS-C; Group B = Severe MIS-C. CK-MB: Creatine kinase-M; NT-proBNP: N terminal-proBrain type natriuretic peptide. * *p* < 0.05 and ^#^ *p* < 0.01, statistically significant difference compared to the control group (one-way ANOVA followed by Tukey’s post-test).

**Figure 3:**
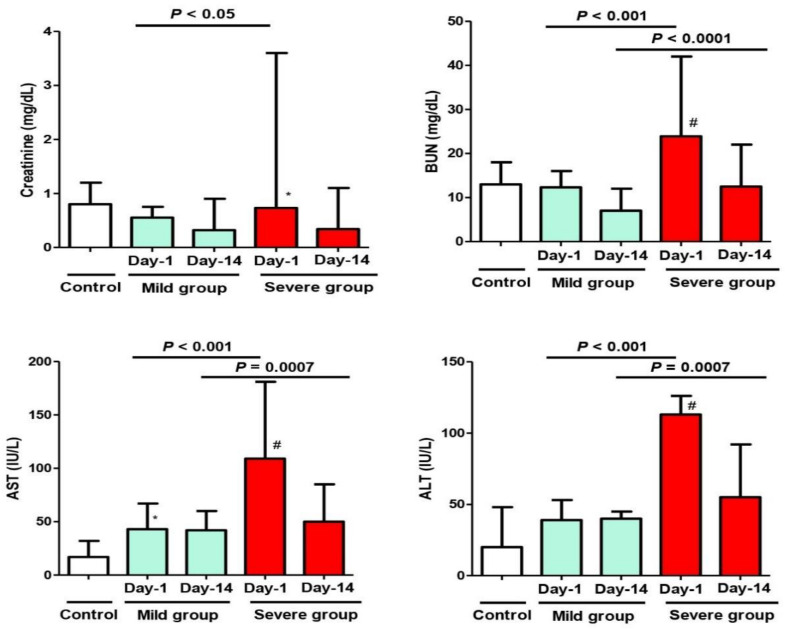
Changes in the biochemical parameters (Creatinine, BUN, AST, ALT) of the study cohort on Day 1 and Day 14 after admission with MIS-C manifestations. Control Group = Simple COVID-19 infection; Group A = Mild MIS-C; Group B = Severe MIS-C. BUN: Blood Urea Nitrogen; ALT: alanine aminotransferase; AST: aspartate aminotransferase. * *p* < 0.05 and ^#^ *p* < 0.01, statistically significant difference compared to the control group (one-way ANOVA followed by Tukey’s post-test).

On Day 1 of admission, there was a significant difference regarding the inflammatory markers (ESR, CRP, serum Ferritin), cardiac injury marker (CK-MB), and biochemical marker (AST) when comparing the mild MIS-C group to the control group (*p* < 0.05). On the other hand, there was an extremely significant difference regarding all the inflammatory markers, all the cardiac injury markers, and all biochemical markers when comparing the severe MIS-C group to the control group (*p* < 0.01). The measured markers were highly abnormal and indicated acute inflammation—of varying degrees—in both study groups, regardless of the clinical presentation (mild or severe) (Figure 1, Figure 2 and Figure 3). When comparing these variables between the phenotypes of the mild group (A) and severe group (B) on Day 1 of admission, there were more elevated values in the latter group, with statistical significance (*p* < 0.05) (Figure 3).

On Day 14 of admission, although all previous abnormal markers in both study groups returned to normal or near normal levels in correlation with improved clinical conditions of the patients, a statistically significant difference between the two groups of patients (mild and severe) was still observed, except for the CRP and serum creatinine levels (Figure 1 and Figure 3)

Additionally, there was a significant difference when comparing the value of any variable in the same MIS-C group between Day 1 and Day 14 (after improvement).

A detailed list of all laboratory investigations on Day 1 after admission is included in Table 2; Day 14 after admission is included in Table 3.

### 3.3. Cytokine Analysis

Regarding cytokines on Day 1, there was a significant difference in their serum levels when comparing the mild MIS-C group to the control (IFN-α, IFN-γ, IL-6, IL-8, IL-10, and CXCL10). On the other hand, there was an extremely significant difference regarding all measured cytokines when comparing the severe MIS-C group to the control group (*p* < 0.01); also, when comparing both study groups (mild and severe), the severe group showed an extremely significant elevation in the following cytokines: IFN-α, IL-1β, IL-6, GM-CSF, and HMGB1 (*p* < 0.0001). Moreover, a less significant increase (*p* < 0.05) was noticed in IL-8, TNF-α, and G-CSF.

**Figure 4:**
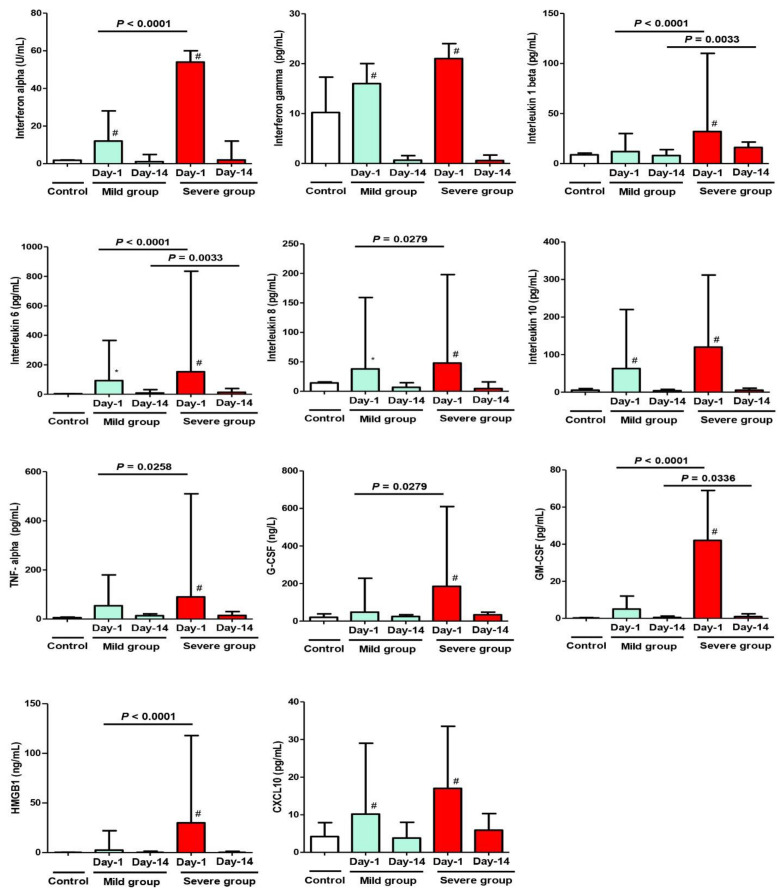
Cytokine analysis at Day 1 and Day 14 after admission with MIS-C manifestations. Control Group = Simple COVID-19 infection; Group A = Mild MIS-C; Group B = Severe MIS-C. * *p* < 0.05 and ^#^ *p* < 0.01, statistically significant difference compared to the control group (one-way ANOVA followed by Tukey’s post-test).

On Day 14, despite the clinical improvement in both groups, some cytokines (IL-1β, IL-6, and GM-CSF) showed persistent significant elevation in the severe group (B) compared to the mild group (A) (*p* < 0.01; see Figure 4). Further, there was a significant difference when comparing the value of any variable in the same group between Day 1 and Day 14 (after improvement).

Table 4 shows the assay for cytokines on Day 1 of admission for the whole cohort, and Table 5 show the assay for cytokines on Day 14 of follow-up in both study groups (mild and severe MIS-C).

### 3.4. Follow-Up and Patients’ Outcome

The clinical course was favorable for most of the study population; all controls (*n* = 30) were discharged on the same day of diagnosis (with simple COVID-19) after they were thoroughly evaluated (clinically and laboratory) to ensure that they do not have any MIS-C manifestations, and none of them reported any MIS-C manifestation within the following two weeks of discharge. For the children diagnosed with MIS-C manifestations (*n*= 60), fifty-six children (93.3%) were discharged from the hospital with no sequelae (with an average hospital/± PICU stay = 7.6 ± 2.3 days); two children (3.3%) were discharged with cardiac sequelae (coronary aneurysm). Unfortunately, there were two children (3.3%) who died following inclusion. One of them was a 2.5-year-old girl who had a 4-day history of fever and presented with severe respiratory distress and shock. She needed MV on the day of admission due to hemodynamic instability and was managed with fluid resuscitation and vasoactive support; she stayed in the ICU for 10 days and died from acute respiratory distress syndrome (ARDS). The other child was a 4-year-old boy who was admitted with fever and symptoms of congestive heart failure; his echocardiography revealed aneurysmal dilatation of the left coronary artery. He needed MV and was managed with IVIG, a high dose of methylprednisolone, and anti-failure measures. Unfortunately, he died after 2 weeks of admission due to refractory reduced systolic function of the heart.

## 4. Discussion

Cytokines are essential mediators of the inflammatory response during the SARS-CoV-2 infection [16]. MIS-C is a recently described clinical entity that can be life threatening in a pediatric population, following peaks of community infection and hospitalizations due to COVID-19 [15,18]. It represents a diagnostic and management challenge, given its similarity to other severe and highly lethal conditions in childhood [17].

In our study, the clinical manifestations of the MIS-C children (including fever, conjunctivitis, respiratory, and gastrointestinal symptoms) were presented more frequently compared to other reports [19,20,21]. Additionally, the percentage of MIS-C children presenting with shock (36.6%), who later required admission to PICU and needed MV, was higher than that in a previous study (only 20%) [22]. On the other hand, the control group showed the classic manifestations of simple COVID-19 infection as reported in the literature [6,11,14,20].

All children admitted to the PICU due to MIS-C-associated COVID-19 infection were previously healthy with no history of co-morbidities, suggesting that this would be the most severe clinical manifestation of SARS-CoV-2 infection in the pediatric age, and this was reported in multiple studies [14,15,23].

Our data demonstrated that about 45% of patients with MIS-C (Group B) required PICU support, mechanical ventilation, and advanced monitoring, with a relatively long stay in hospital/PICU (10.5 ± 2.2 days). This was inconsistent with reports from France, USA, and Chile, where intensive management was required for 70–80% of these patients with a shorter hospital/PICU stay (5.2 ± 2.5) [14,17,18]. This discrepancy might be due to different variants of SARS-CoV-2 that may affect the inflammatory response influenced by the virus genotype. Recent reports discussed and confirmed the presence of a discrepancy regarding the clinical severity and the prognosis according to the genotype of the virus variant [24,25]. Unfortunately, the study was conducted in a center that does not have the facility to detect the genotype of the SARS-CoV-2 variants.

The hematologic aspect of the disease (either simple COVID-19 or MIS-C) is characterized by anemia, leukocytosis, lymphopenia, and normal or decreased platelet counts [1,4,14,21]. The other laboratory findings in the MIS-C groups (mild and severe) were characterized by elevated systemic inflammatory markers; i.e., increased ESR, CRP, ferritin, and LDH values, which are consistent with the findings among other patients with MIS-C in multiple studies [9,12,15,20,21,22,23,26,27]. On the other hand, the control group (simple COVID-19) did not show abnormal serum levels of these inflammatory markers (ESR, CRP, serum Ferritin, and LDH) as reported previously [1,4,21]. These data suggest that the elevated laboratory parameters indicated a strong inflammatory status. Therefore, during the COVID-19 pandemic, the differential diagnosis of severe systemic inflammatory/infectious conditions should consider MIS-C among its probable causes [21,28,29,30].

Our results, in concordance with other studies, confirmed the positive correlation between elevated inflammatory markers (mainly ESR, CRP, ferritin, and LDH in the severe group) with the clinical manifestation of MIS-C patients, showing their remarkable role in the short-term outcomes [31,32].

In the present study, the elevation of indices of cardiac injury is consistent with the findings of other studies, which reported that MIS-C is mainly associated with cardiac complications (in 70–80% of patients) [33]. Through the comparison of the cardiac injury markers between the control group and MIS-C groups (mild and severe), it was found that the control group did not show any abnormal levels of cardiac injury markers, in congruence with other studies [30,31,34]. Of note, all cardiac injury markers of severe MIS-C children were found significantly higher than those of mild ones in the present study, a finding that might suggest their important role in the disease severity and prognosis in children, as supported by studies that reported an increase in cardiac injury markers may be related to poor prognosis [34,35]. However, during the follow-up of our patients, these cardiac injury markers dropped to their normal levels after 2 weeks of hospitalization and management, consistent with the results documented by other related reports [15,31,36].

In agreement with a report by Henry et al., the control group showed normal cytokines level [4]; in turn, the cytokines (IL-1β, IL-6, IFN-α, GM-CSF, and HMGB1) on Day 1 of presentation showed significant upregulation, especially in the severe group (B), which confirmed their role as the main etiological factor affecting short-term outcomes in MIS-C patients. Other cytokines (IL-8, TNF-α, and G-CSF) were expressed at slightly more than normal serum levels, indicating their secondary role in the manifestations of MIS-C. On Day 14, when comparing values in both groups (mild Vs severe), some cytokines (IL-1β, IL-6, and GM-CSF) were persistently elevated, especially in Group B, confirming their role in the severity of manifestations, disease progression, and their effect on mid-term outcomes. Elevated immunological markers (mainly IL-6, IL-1β) were significantly associated with increased odds of severe disease progression, the length of hospital/PICU stay, and correlating positively to the clinical phenomena, as proved by several international studies [32,37,38,39,40].

Moreover, high levels of interferons in our study (especially in the severe group) are inconsistent with the findings of a study, which reported that MIS-C patients might have impaired interferon activity (IFN-α, IFN-γ) [41]. However, by following-up our patients, we observed a drop in the interferon activity on Day 14 of presentation, with improvement in clinical manifestations and discharge of patients.

Following international reports and the local multidisciplinary approach, supportive measures were used for the management of mild cases, while severe MIS-C cases were administered IVIG and corticosteroids. Twenty percent of the severe cases continue to have persistent fever despite the use of the first dose of IVIG and corticosteroids; they required another dose of IVIG and an increase of more than four times the initial dose of corticosteroids for fever resolution. Thus, we could recommend administration of the highest dose of methylprednisolone (10 mg/kg/day) on admission of patients diagnosed with severe MIS-C; this could eventually resolve fever, decrease the intensive care support, and shorten the disease duration, as previously reported [16,17].

Our study has limitations. First, the low number of patients did not allow us to establish associations between the variables studied. Second, our investigation was conducted at a single center and with a specific racial/ethnicity composition. Third, the absence of the genotype data of the SARS-CoV-2 variants. Future large-scale studies from multiple centers examining the MIS-C population are warranted to understand this disease further and overcome these limits; however, this is one of the earliest studies to evaluate the inflammatory response of MIS-C among children in Saudi Arabia.

## 5. Conclusions

MIS-C associated with SARS-CoV-2 infection is characterized by acute elevation of inflammatory markers, tissue injury markers, and cytokines (mainly IL-1β, IL-6, IFN-α, GM-CSF, and HMGB1, and less significantly increased IL-8, TNF-α, and G-CSF). At midterm follow-up, the elevated cytokines reported were IL-1β, IL-6, and GM-CSF, mainly in the severe group, which might explain the clinical phenomena. These findings could be correlated with short- and mid-term outcomes of the MIS-C patients.

## Figures and Tables

**Table 1:** Demographics, clinical presentations, and basic laboratory workup of the study cohort and controls at Day 1 of admission.

Variable	Control Group (Simple COVID-19) (*n* = 30)	Group A (Mild MIS-C) (*n* = 33)	Group B (Severe MIS-C) (*n* = 27)	*p* Value ^$^
**Age** (years)	6.8 ± 4.1	7.2 ± 4.1	6.1 ± 4.8	0.6199
**Sex** -Male -Female	17 (56.6%) 13 (43.3%)	15 (45.5%) 18 (54.5%)	16 (59.3%) 11 (40.7%)	0.1552
**Ethnicity** -Saudi -Non-Saudi	20 (66.7%) 10 (33.3%)	20 (60.6%) 13 (39.4%)	18 (66.7%) 9 (33.3%)	0.3923
**Clinical presentations** Fever Respiratory Gastrointestinal Rash Shock Conjunctivitis Lymphadenopathy	25 (83.3%) 17 (56.6%) 16 (53.3%) 4 (13.3%) 0 (0%) 23 (76.6%) 2 (6.6%)	33 (100%) 23 (69.7%) * 27 (81.8%) * 5 (15.2%) 0 (0%) 25 (75.8%) 3 (9.1%)	27 (100%) 27 (100%) ^#,$^ 21 (77.7%) * 4 (14.8%) 22 (81.5%) ^#,$^ 23 (85.2%) 2 (7.4%)	1.0000 **0.0011 ^$^** 0.7357 0.7928 **<0.0001 ^$^** 0.3143 0.7237
**Weight** (kg)	9.53 ± 3.1	10.52 ± 3.9	8.87 ± 2.9	0.0734
**Heart rate** (beats/min)	110.5 ± 9.23	113.43 ± 12.14	122.6 ± 15.93 ^#,$^	**0.05 ^$^**
**Respiratory rate** (breaths/min)	28.14 ± 9.8	34.23 ± 8.14	47.96 ± 11.58 ^#,$^	**<0.0001 ^$^**
**Duration between SARS-CoV-2 infection and MIS-C diagnosis** (days)	-	6.1 ± 2.4	4.5 ± 1.7	**0.002 ^$^**
**Length of hospital/± PICU stay** (days)	-	3.8 ± 1.3 (Range: 3–6)	15.5 ± 2.8 (Range: 6–17.4)	**<0.0001 ^$^**
**Hematology (median and IQR)**				
**Hemoglobin** (gm/dL) (reference, 11.5–15.6)	11.6 (9.2–13.3)	10.6 (8.4–12.9)	9.9 (8.6–10.9) ^#^	0.4666
**WBC count** (10^9^/L) (reference, 4.5–13.5)	11.04 (5.2–13.1)	14.4 (6.1–20.1) *	18.3 (14.3– 20.2) ^#,$^	**<0.01 ^$^**
**Lymphocytes** (10^9^/L) (reference, 2–10)	1.2 (0.9–2.3)	0.8 (0.6–1.7) *	0.8 (0.7–0.9) ^#^	0.9941
**Platelet count** (10^9^/L) (reference, 140–450)	155 (145–490)	165 (112–247) ^#^	139 (114–246) ^#^	0.4666
**INR** (seconds) (reference, ≤ 1.1)	0.7 (0.4–1.0)	0.8 (0.2–1.1)	1.2 (0.9–1.3) ^#,$^	**<0.001** ^$^

* *p* < 0.05 and ^#^ *p* < 0.01, statistically significant difference compared to the control group; ^$^ *p* < 0.05, statistically significant difference between the mild and severe groups (one-way ANOVA followed by Tukey’s post-test).

**Table 2:** Inflammatory markers, cardiac injury markers, and biochemical workup of the study cohort on Day 1 after admission with MIS-C manifestations.

Variable	Control Group (*n* = 30) Median (IQR)	Group A (Mild) (*n* = 33) Median (IQR)	Group B (Severe) (*n* = 27) Median (IQR)	*p* Value ^$^
Inflammatory Markers				
**ESR** (mm/hr) (reference, 1–20)	5 (3–18)	38 (20–54) *	52 (38–63) ^#,$^	<0.001 ^$^
**CRP** (mg/dL) (reference, ≤5)	4 (2–10)	5.8 (3.14–36.15) *	26 (13–31) ^#^	<0.05 ^$^
**Ferritin** (ng/mL) (reference, ≤150)	85 (70–150)	220 (160–402) *	477 (281–980) ^#,$^	<0.001 ^$^
**Lactate dehydrogenase** (IU/L) (reference, 120–260)	170 (140–260)	345 (265–492)	369 (287–580) ^#,$^	<0.001 ^$^
**Cardiac markers**				
**CK-MB** (IU/L) (reference, ≤25)	15.2 (5.8–23.9)	28.7 (24.8–34) *	73.7 (30.6–78) ^#,$^	0.0002 ^$^
**Troponin-T** (ng/mL) (reference, 20–60)	35 (25–65)	25 (17–52)	54 (35–398) ^#,$^	<0.0001 ^$^
**NT-proBNP** (pg/mL) (reference, <300)	90 (145–290)	154 (215–431)	784 (631–1135) ^#,$^	<0.0001 ^$^
**Biochemistry**				
**Creatinine** (mg/dL) (reference, 0.6–1.1)	0.8 (0.5–1.2)	0.55 (0.42–0.75)	0.73 (0.2–3.6) *^,^^$^	<0.05 ^$^
**BUN** (mg/dL) (reference, 8–20)	13 (9–18)	12.3 (9–16)	23.9 (20–42) ^#,$^	<0.001 ^$^
**AST** (IU/L) (reference, 10–37)	17 (12–32)	43 (28–67) *	109 (76–181) ^#,$^	<0.001 ^$^
**ALT** (IU/L) (reference, 16–61)	20 (15–48)	39 (17–53)	113 (46–126) ^#,$^	<0.001 ^$^

ESR: Erythrocyte sedimentation rate; CRP: C-reactive protein; LDH: Lactate dehydrogenase; BUN: Blood Urea Nitrogen; ALT: alanine aminotransferase; AST: aspartate aminotransferase. * *p* < 0.05 and ^#^ *p* < 0.01, statistically significant difference compared to the control group; ^$^ *p* < 0.05, statistically significant difference between the mild and severe groups (one-way ANOVA followed by Tukey’s post-test).

**Table 3:** Inflammatory markers, cardiac injury markers, and biochemical workup of the study cohort on Day 14 after admission with MIS-C manifestations.

Variable	Group A (Mild) (*n* = 33) Median (IQR)	Group B (Severe) (*n* = 27) Median (IQR)	*p* Value ^#^
Inflammatory Markers			
**ESR (mm/hr)** (reference, 1–20)	8 (6–15)	12 (10–18)	0.0002 ^#^
**CRP** (mg/dL) (reference, ≤5)	2 (1–5)	4 (0–8)	0.4666
**Ferritin** (ng/mL) (reference, ≤150)	60 (20–98)	90 (30–200)	0.0279 ^#^
**LDH** (IU/L) (reference, 120–260)	238 (170–280)	250 (200–320)	0.0279 ^#^
**Cardiac markers**			
**CK-MB** (IU/L) (reference, ≤25)	18 (11–25)	25 (15–33)	0.0033 ^#^
**Troponin-T** (ng/mL) (reference, 20–60)	15 (9–48)	17 (15–63)	0.0033 ^#^
**NT-proBNP** (pg/mL) (reference, <300)	107 (60–134)	280 (200–410)	<0.0001 ^#^
**Biochemistry**			
**Creatinine** (mg/dL) (reference, 0.6–1.1)	0.32 (0.2–0.9)	0.34 (0.2–1.1)	0.0625
**BUN** (mg/dL) (reference, 8–20)	7 (4.5–12)	12.5 (10–22)	<0.0001 ^#^
**AST** (IU/L) (reference, 10–37)	42 (27–60)	50 (46–85)	0.0007 ^#^
**ALT** (IU/L) (reference, 16–61)	40 (15–45)	55 (36–92)	0.0007 ^#^

ESR: Erythrocyte sedimentation rate; CRP: C-reactive protein; LDH: Lactate dehydrogenase; BUN: Blood Urea Nitrogen; ALT: alanine aminotransferase; AST: aspartate aminotransferase. ^#^ *p* < 0.05, statistically significant difference between the mild and severe groups (Mann–Whitney U test for the median and inter quartile range).

**Table 4:** Assay of cytokines on Day 1 after admission with MIS-C manifestations.

Variable	Control Group (*n* = 30) Median (IQR)	Group A (Mild) (*n* = 33) Median (IQR)	Group B (Severe) (*n* = 27) Median (IQR)	*p* Value ^$^
**Interferon alpha** (IFN-α) (reference, <2 U/mL)	1.8 (0.5–2)	12 (8–28) ^#^	54 (18–60) ^#,$^	<0.0001 ^$^
**Interferon gamma** (IFN-γ) (reference, 0.10–18.00 pg/mL)	10.2 (1.9–17.3)	16 (14–20) ^#^	21 (15–24) ^#^	0.9940
**Interleukin 1 beta** (IL-1β) (reference, 0.5–12 pg/mL)	8.7 (1.6–10.4)	12 (5–30)	32 (18–110) ^#,$^	<0.0001 ^$^
**Interleukin 6** (IL-6) (reference, <5 pg/mL)	3.8 (0.98–4.87)	93 (44–365) *	153 (69–835) ^#,$^	<0.0001 ^$^
**Interleukin 8** (IL-8) (reference, 2.8–17.0 pg/mL)	14.3 (4.7–15.9)	38 (25–159) *	48 (32–198) ^#,$^	0.0279 ^$^
**Interleukin 10** (IL-10) (reference, <10 pg/mL)	5.2 (0.9–9.6)	63 (43–220) ^#^	120 (40–312) ^#^	0.4666
**Tumor necrosis factor alpha** (TNF-α) (reference, <8.5 pg/mL)	5.9 (4.1–8.2)	54 (42–180)	90 (74–510) ^#,$^	0.0258 ^$^
**Granulocyte colony stimulating factor** (G-CSF) (reference, 5–42 ng/L)	19.8 (7.2–38.8)	48 (15–228)	185 (70–610) ^#,$^	0.0279 ^$^
**Granulocyte-macrophage colony-stimulating factor** (GM-CSF) (reference, 0–0.39 pg/mL)	0.2 (0.12–0.4)	5 (0.4–12)	42 (5–69) ^#,$^	<0.0001 ^$^
**high-mobility group box 1** (HMGB1) (reference, 0.2–0.4 ng/mL)	0.35 (0.15–0.39)	2.5 (0.9–22)	30 (15–118) ^#,$^	<0.0001 ^$^
**Human C-X-C motif chemokine ligand 10** (CXCL10) (reference, <7.8 pg/mL)	4.2 (1.9–7.9)	10.2 (6–29) ^#^	17 (6.9–33.5) ^#^	0.0792

* *p* < 0.05 and ^#^ *p* < 0.01, statistically significant difference compared to the control group; ^$^ *p* < 0.05, statistically significant difference between the mild and severe groups (one-way ANOVA followed by Tukey’s post-test).

**Table 5:** Assay of cytokines on Day 14 after admission with MIS-C manifestations.

Variable	Group A (Mild) (*n* = 33) Median (IQR)	Group B (Severe) (*n* = 27) Median (IQR)	*p* Value ^#^
**Interferon alpha** (IFN-α) (reference, <2 U/mL)	1.1 (0.8–4.9)	2 (0.5–12)	0.4666
**Interferon gamma** (IFN-γ) (reference, 0.10–18.00 pg/mL)	0.66 (0.11–1.57)	0.58 (0.11–1.69)	0.9791
**Interleukin 1 beta** (IL-1β) (reference, 0.5–12 pg/mL)	8 (5–14)	16 (5–21.5)	0.0033 ^#^
**Interleukin 6** (IL-6) (reference, <5 pg/mL)	9 (8–32)	13 (9–39)	0.0033 ^#^
**Interleukin 8** (IL-8) (reference, 2.8–17.0 pg/mL)	6.8 (2.5–14.6)	4.7 (2–16)	0.5194
**Interleukin 10** (IL-10) (reference, <10 pg/mL)	3.82 (2.17–7.27)	5.23 (3.31–10.64)	0.425
**Tumor necrosis factor alpha** (TNF-α) (reference, <8.5 pg/mL)	13.5 (8.2–21.4)	15 (8.1–30)	0.5194
**Granulocyte colony stimulating factor** (G-CSF) (reference, 5–42 ng/L)	24.3 (7.9–34)	33.5 (21.3–48)	0.0508
**Granulocyte-macrophage colony-stimulating factor** (GM-CSF) (reference, 0–0.39 pg/mL)	0.45 (0.1–1.2)	0.9 (0.3–2.5)	0.0336 ^#^
**high-mobility group box 1** (HMGB1) (reference, 0.2–0.4 ng/mL)	0.3 (0.1–1.4)	0.3 (0.2–1.3)	0.9935
**Human C-X-C motif chemokine ligand 10** (CXCL10) (reference, <7.8 pg/mL)	3.8 (1.2–8)	5.9 (4.7–10.3)	0.9924

^#^ *p* < 0.05 statistically significant difference between the mild and severe groups (Mann–Whitney U test for the median and inter quartile range).

## Data Availability

Not applicable.
